# Butyrate suppresses experimental necrotizing enterocolitis–induced brain injury in mice

**DOI:** 10.3389/fped.2023.1284085

**Published:** 2023-12-07

**Authors:** Maribel Martinez, Wei Yu, Heather L. Menden, Tianhua Lei, Paula Monaghan-Nichols, Venkatesh Sampath

**Affiliations:** ^1^ Division of Neonatology, Department of Pediatrics, Children’s Mercy Kansas City, Kansas, MO, United States; ^2^ Neonatal Diseases Research Program, Children’s Mercy Research Institute, Children’s Mercy Kansas City, Kansas, MO, United States; ^3^ Department of Biomedical Sciences, University of Missouri Kansas City School of Medicine, Kansas, MO, United States

**Keywords:** butyrate, NEC, brain injury, inflammation, microglia, oligodendrocyte

## Abstract

**Background:**

Necrotizing enterocolitis (NEC) is a devastating disease in premature infants, and 50% of infants with surgical NEC develop neurodevelopmental defects. The mechanisms by which NEC-induced cytokine release and activation of inflammatory cells in the brain mediate neuronal injury, and whether enteral immunotherapy attenuates NEC-associated brain injury remain understudied. Based on our prior work, which demonstrated that experimental NEC-like intestinal injury is attenuated by the short-chain fatty acid, butyrate, in this study, we hypothesize that NEC-induced brain injury would be suppressed by enteral butyrate supplementation.

**Methods:**

A standardized NEC mouse model [enteral formula feeding, lipopolysaccharide (LPS), and hypoxia] was used. Mice were randomized into the following groups: control, NEC, butyrate pretreated NEC, and butyrate control. NEC scoring (1–4 with 4 representing severe injury) was performed on ileal sections using a validated scoring system. Intestinal and brain lysates were used to assess inflammation, proinflammatory signaling, and apoptosis.

**Results:**

NEC-induced intestinal injury was attenuated by butyrate supplementation. NEC-induced microglial activation in the cerebral cortex and hippocampus was suppressed with butyrate. NEC increased the number of activated microglial cells but decreased the number of oligodendrocytes. Butyrate pretreatment attenuated these changes. Increased activation of proinflammatory Toll-like receptor signaling, cytokine expression, and induction of GFAP and IBA1 in the cerebral cortex observed with NEC was suppressed with butyrate.

**Conclusion:**

Experimental NEC induced inflammation and activation of microglia in several regions of the brain, most prominently in the cortex. NEC-induced neuroinflammation was suppressed with butyrate pretreatment. The addition of short-chain fatty acids to diet may be used to attenuate NEC-induced intestinal injury and neuroinflammation in preterm infants.

## Introduction

Necrotizing enterocolitis (NEC), characterized by acute intestinal inflammation and necrosis, remains a major cause of neonatal morbidity and mortality in premature infants. The pathogenesis of NEC involves a combination of factors such as prematurity, formula feeding, gut ischemia, genetic predisposition, and intestinal dysbiosis. Abnormal activation of the intestinal Toll-like receptor 4 (TLR4), an innate immune receptor recognizing Gram-negative bacteria, results in intestinal epithelial cell death and the translocation of bacteria from the gut into the circulation (1, 2). Of the surviving infants, more than half go on to show evidence of significant neurodevelopmental impairment (3–5). Shah et al. noted that infants with NEC experienced changes in the cerebral cortex and white matter regions when compared with non-NEC infants (6). Mechanistic insights into the cellular and molecular pathways mediating NEC-induced brain injury are poorly defined. Intestinal injury and subsequent activation of the systemic inflammatory response after NEC have been linked to the activation of cell death cascades in microglia and other brain cell types. These neuro-inflammatory changes and cellular injury are supposed to contribute to cognitive impairment and cerebral palsy seen in NEC survivors (4, 5, 7).

While studies evaluating the mechanisms by which NEC causes brain injury and impairs development have been limited, there is clear experimental evidence linking NEC to brain injury. Sun et al. showed that experimental NEC was associated with acute injury in the hippocampal region (8). Similarly, Niño et al. noted significant impairment in myelination and microglial activation in the brain of a mouse with NEC (9, 10). However, a more complete understanding of the pathways involved in NEC-induced brain injury and inflammation response is essential for identifying therapies that can mitigate the consequences of neonatal brain injury. In this study, we investigate whether enteral therapy with butyrate, a naturally occurring product of bacterial fermentation in the intestine, can rescue NEC-induced brain injury.

Investigations into the therapeutic potential of butyrate, a short-chain fatty acid (SCFA) produced by intestinal flora, has shown great promise for NEC-induced intestinal injury (11–13). Both our laboratory and others have shown that SCFAs such as butyrate repress NEC-induced inflammation and gut injury through several mechanisms, including the promotion of intestinal epithelial barrier function and activation of single immunoglobulin interleukin-1 related receptor (SIGIRR), a known inhibitor of TLR-mediated inflammation (2, 11, 14, 15). A mechanistic understanding of how butyrate affects the inflammatory gut–brain axis is valuable for designing effective strategies to prevent NEC. We hypothesized that SCFAs will reduce NEC-induced brain injury by inhibiting inflammation and cell death. To test our hypothesis, we evaluated the effects of enteral butyrate supplementation on experimental NEC-induced neonatal brain injury in mouse pups.

## Methods

### Animal model

All studies were performed at the University of Missouri School of Medicine. Pregnant C57BL/6 mice were obtained from Charles River. Litters of mice were breast-fed by the mother until days of life (DOL) 7, separated from the mother, and randomly assigned to either the control, NEC, NEC plus butyrate, or butyrate control groups. NEC was induced using a standard NEC protocol (14) (see Supplementary Material 3). The mice were gavage-fed with a 24-Fr angiocatheter for five times in a 24 h period. Three of the feeds received 0.15 ml every 3 h with a formula (15 g Similac 60/40 in 75 ml Esbilac canine milk replacer), and two of the feeds received mixed 0.15 ml every 3 h with a formula with lipopolysaccharide *Escherichia coli* 055:B5 (200 μl/5 g of mouse body weight, Sigma, St Louis, MO, USA). The mice were simultaneously exposed to hypoxic stress (5% oxygen and 95% nitrogen) twice daily for 10 min each of exposure. *This model represents a reproducible model of mucosal injury and systemic inflammation in the postnatal period*. Prior to NEC induction, the NEC plus butyrate group and butyrate control group received 10 µl of 60 mM butyrate twice a day on day of life 5 and 6; this dose was chosen on the basis of butyrate concentration in the human intestine lumen and previously reported research findings (11, 15–17). Given that butyrate has a short half-life (18), in addition to pretreatment with butyrate as noted above, formula milk was supplemented with 60 mM butyrate in the NEC butyrate group. After 3 days of NEC induction, the mice were euthanized on DOL-10 using an intraperitoneal injection of phenobarbital, and the terminal ileum and brain were harvested. The control and butyrate control pups were also sacrificed on DOL-10 for comparison with the NEC group. Brain and ileum were collected and divided into two parts: formalin for staining and RNA later for quantitative polymerase chain reaction (qRT-PCR).

### Brain tissue collection

Brain tissue was collected and divided into two equal parts (1) half of the brain was placed in Carnoy's (30% chloroform, 60% ethanol, 10% glacial acetic acid), embedded in paraffin blocks, and sectioned at 5 µm. Specific brain regions including the (1) cortex, (2) hippocampus, and (3) cerebellum were dissected and used for RNA and Western blot analysis.

### Hematoxylin and Eosin staining to evaluate the histologic grading of NEC

Histologic slides were stained with hematoxylin and eosin. Slides were scanned using a Leica Biosystems Slide Scanner. A standardized 4-point scale as previously described was used to grade intestinal injury in the ileum (19), by two separate investigators, one of whom was blinded to the experimental conditions (MM and WY). Briefly, injuries are ranked on a scale of 0–4: 0, healthy mucosa; 0 = normal intestine; 1 = disarrangement of villus enterocytes, villus-core separation; 2 = significant disarrangement of villus enterocytes, villus-core separation down the sides of villi, blunting of villi; 3 = epithelial sloughing of villi, loss of villi; 4 = intestinal necrosis or perforation. A score of 2 or higher indicated the development of experimental NEC. The analysis was completed using the system's imaging software (Aperio Imagescope). One of the two personnel who assessed intestinal injury scores was blinded to treatment groups.

### Quantification of inflammatory cytokine mRNA expression using real-time PCR

Pieces of the cortex, cerebellum, and hypothalamus were homogenized using a bullet blender (Midwest Scientific, St Louis, MO, USA). Total RNA was extracted using the Trizol standard protocol (Thermo Fisher, Waltham, MA, USA), and cDNA was synthesized from 1 μg of RNA using the iScript cDNA synthesis kit (Bio-Rad, Hercules, CA, USA). The transcripts were amplified and gene expression data were collected on a ViiA7 with an SYBR Green master mix. Prevalidated KiCqStart SYBR primers were purchased from Sigma, and the following primers were used: Actin (ACTB), tumor necrosis factor alpha (TNF-α), IL-6, IL-8 (KC), interleukin-1 beta (IL-1β), and TLR4. Actin was used as the housekeeping gene. The relative gene expression of these markers was calculated by the Pfaffl method and delta-delta CT (20).

### Protein expression by Western blotting

To analyze the protein levels in the cortex and cerebellum, protein lysates were obtained from mouse brain tissues after being extracted using a radioimmunoprecipitation assay (RIPA) lysis buffer containing commercially available Halt protease and phosphatase inhibitors (Thermo Fisher) that were homogenized by using the bullet blender (Midwest Scientific). The concentration of purified lysates was measured using a bicinchoninic acid Protein Assay Kit (Thermo Fisher). Equal amounts of lysates were loaded and separated by sodium dodecyl sulfate–polyacrylamide gel electrophoresis, and gels were blotted to polyvinylidene difluoride membranes. The membranes were incubated with primary antibodies, washed, and again incubated with peroxidase-conjugated secondary antibodies (Abcam, Waltham, MA, USA). The primary antibodies are as follows: Rabbit anti-Phospho-p65 (Ser536, Cell Signaling Technologies, CST, Danvers, MA, USA), rabbit anti-p65 (CST), rabbit anti-Phospho-p38 (Thr180/Tyr182, CST), rabbit anti-p38 (CST), rabbit anti-TLR4 (Abcam), rabbit anti-SIGIRR (Thermo Fisher), mouse anti-GFAP (CST), rabbit anti-IL6 (CST), rabbit anti-Cleaved Caspase 3 (CC3, CST), rabbit anti-IBA1 (Abcam), mouse anti-ICAM1 (SCBT, Santa Cruz, CA, USA), and mouse anti-β-Actin (ACTB, Sigma). Reactive proteins were detected using a chemiluminescence detection system and visualized on the imaging system iBright FL1000 (Thermo Fisher). Densitometry was performed using ImageJ software (NIH, Bethesda, MD, USA), and changes were normalized to beta-actin (ACTB) or the corresponding non-phosphorylated antibody.

### Immunofluorescence for astrocytes, glial cells, and cell proliferation

A segment of the cortex, cerebellum, and hypothalamus was embedded in paraffin. The paraffin blocks were cut at 5 μm and adhered to positive-charged slides. The samples were air-dried, washed in PBS and PBS-Tween for 5 mins, blocked in 10% heat-inactivated normal goat serum (HINGS, Jackson ImmunoResearch, West Grove, PA, USA), and incubated with the primary antibodies or control overnight at 4°C. Sections were subsequently washed in PBS ×3 and incubated with a secondary antibody for 2 h at room temperature, washed, and counterstained with 4,6-diamidino-2-phenylindole (DAPI; Thermo Fisher). The primary antibodies are as follows: CNPase-2′, 3′-cyclic nucleotide 3′-phosphodiesterase (anti-mouse CNPase, Abcam) to identify myelinating oligodendrocytes; Glial Fibrillar Acidic Protein (anti-mouse GFAP, Cell Signaling) to identify glial cells; ionized calcium-binding adaptor molecule 1 (anti-Rabbit IBA1, Abcam) to identify microglia; Phospho-histone H3 S28 (anti-rabbit Phis, Abcam) to identify replicating cells in the M phase of the cell cycle. The secondary antibodies are as follows: for CNPAse, anti-mouse Alexa Fluor 647 (Abcam); for GFAP, anti-mouse Alexa Fluor 488 (Abcam); for IBA1, anti-rabbit Cyanine 3 (Abcam); for Phis, anti-rabbit Cyanine 3 (Abcam). The nucleus was stained with DAPI. Images were captured using EVOS imaging software. The images were taken at 20× and processed with Adobe Photoshop. The images were imported into the QuPath version 0.2.3 program and analyzed using the “Positive Cell Detection” software (21). Each channel (red, green, and blue) was analyzed separately using the following settings: requested pixel size (0.5 µm), Sigma (1.5 μm), threshold (25), cell expansion (5–15 µm), and mean nuclear or cytoplasmic thresholds per channel were set to (1+:10, 2+:20, 3+:30), the minimum area was set to 10 µm^2^, and the maximum area was set to 400 µm^2^. The parameters measured are as follows: number of cells detected, number of cells positive, centroid in the X and Y axes, number positive per mm, area in µm^2^, and perimeter in µm.

### Data analysis

Data was represented as mean ± SD. For all data, we initially examined whether the distribution of data was Gaussian using the D’Agostino–Pearson omnibus normality test. If data were normally distributed, then ANOVA with Bonferroni correction was used for analysis. If data did not meet Gaussian assumptions, the Kruskal–Wallis test with Dunn's correction was used for analysis. A score of *p* < 0.05 was considered significant for all variables. Animal considerations: each experimental group contained an *n* ≥ 5. RNA quantification and PCR results had two to three technical replicates. Statistical analysis was done using GraphPad Prism v9.0 (San Diego, CA, USA). An *n* of 5 was used for immunofluorescent staining of pup brains; specific regions included the cortex, hippocampus, and cerebellum.

## Results

### Butyrate decreases gut injury in experimental NEC

To evaluate the effects of butyrate administration on intestinal and brain injury in the experimental mouse NEC model, wild-type C57BL/6 pups were fed a combination of formula milk, enteral LPS, and timed hypoxia exposure. We first examined the protective effect of butyrate in the neonatal intestine. Pups with NEC showed epithelial layer sloughing and separation of the submucosal layer and lamina propria (Supplementary Material 1A) when compared with the butyrate-treated group, which had decreased epithelial disruption and an increased amount of villi with normal morphology. A histologic evaluation of the terminal ileum based on pathological damage on a scale of 0 (normal) to 4 (bowel necrosis) showed that the NEC pups had a higher degree of intestinal injury when compared with the control and NEC butyrate groups (NEC mean 2.5 ± 0.4 vs. NEC + butyrate; mean 1.2 ± 0.3, *p* < 0.05, *n* > 5/group). These findings are consistent with previous findings published by our group (11).

### Administration of butyrate attenuates NEC-induced microglial activation and oligodendrocyte loss in the neonatal brain

**Figure 1:**
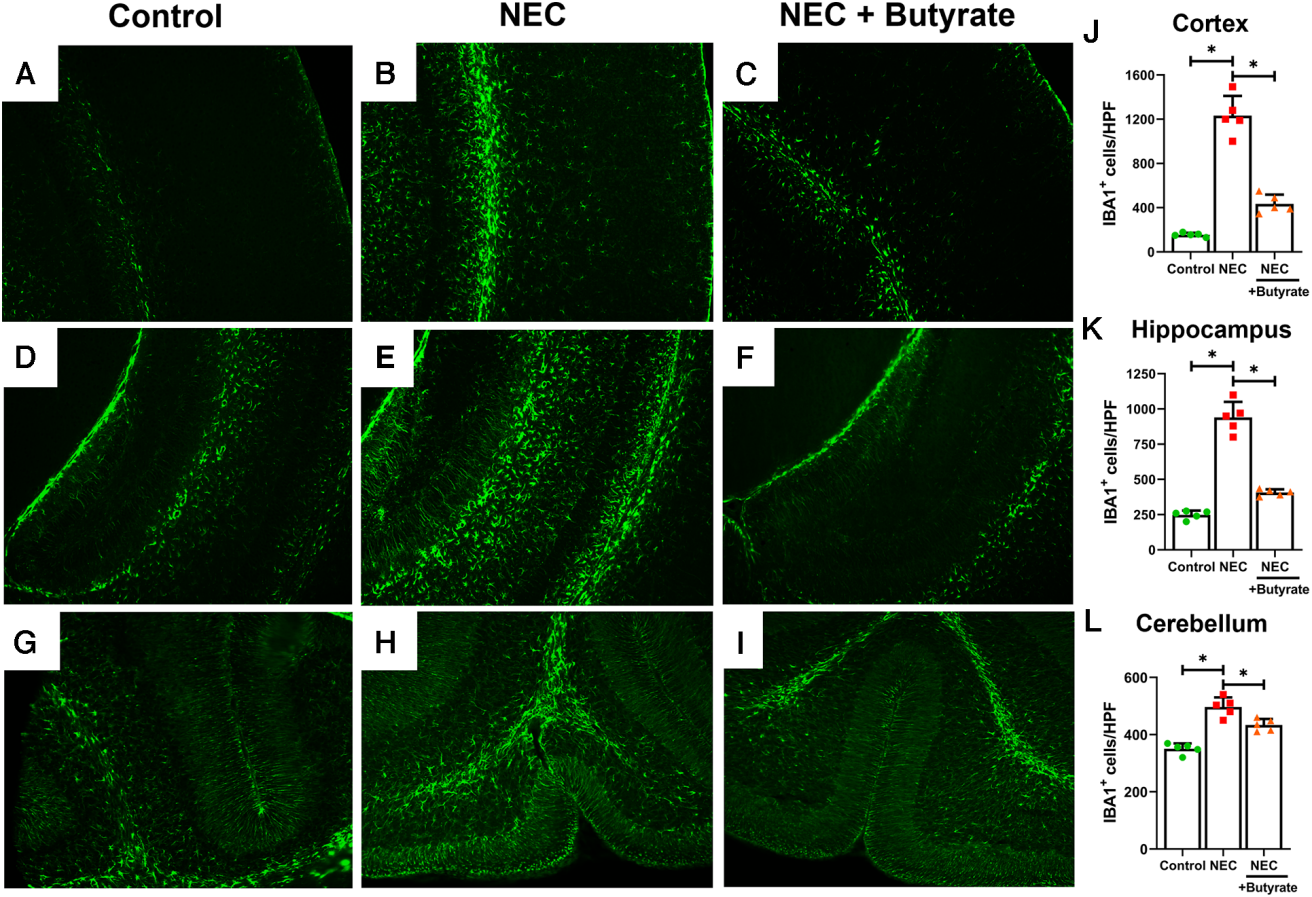
Immunofluorescence studies evaluating brain microglial activation in a region-specific manner. Neonatal mice were treated with enteral butyrate between P5 and P7, following which experimental NEC was induced between P7 and P10. Cortex, cerebellum, and hippocampal sections were used for IBA1 immunofluorescence staining. Three different regions of the brain evaluated for activated microglial cells in the (**A**) mouse control cortex; (**B**) mouse NEC cortex; (**C**) mouse NEC + butyrate cortex; (**D**) mouse control hippocampus; (**E**) mouse NEC hippocampus; (**F**) mouse NEC + butyrate hippocampus; (**G**) mouse control cerebellum; (**H**) mouse NEC cerebellum; (**I**) mouse NEC + butyrate cerebellum. Cell counts for the respective regions comparing the control vs. NEC vs. NEC plus butyrate group: (**J**) cortex; (**K**) hippocampus; and (**L**) cerebellum. High-powered field evaluation (*n* = 5). Data shown as means ± SD, *p < 0.05, one-way ANOVA with Bonferroni correction. Images shown at 10×.

**Figure 2:**
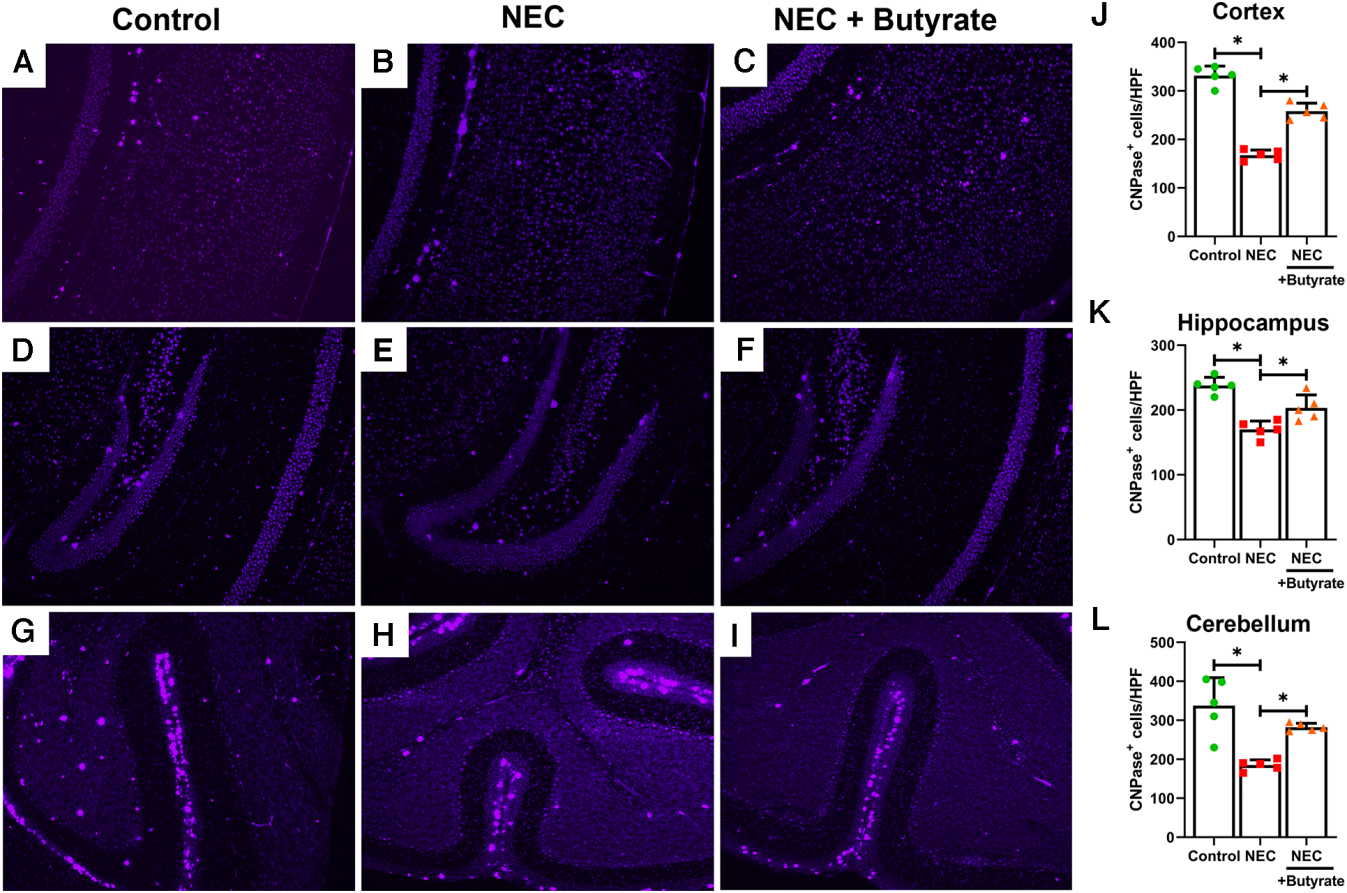
Immunofluorescence studies evaluating oligodendrocyte presence in specific areas of the brain of a mouse. Neonatal mice were treated with enteral butyrate between P5 and P7, following which experimental NEC was induced between P7 and P10. Cortex, cerebellum, and hippocampal sections were used for CNPase immunofluorescence staining. Three different regions of the brain evaluated for oligodendrocytes in the (**A**) mouse control cortex; (**B**) mouse NEC cortex; (**C**) mouse NEC + butyrate cortex; (**D**) mouse control hippocampus; (**E**) mouse NEC hippocampus; (**F**) mouse NEC + butyrate hippocampus; (**G**) mouse control cerebellum; (**H**) mouse NEC cerebellum; (**I**) mouse NEC + butyrate cerebellum. Cell counts for the respective regions comparing the control vs. NEC vs. NEC plus butyrate group: (**J**) cortex; (**K**) hippocampus; and (**L**) cerebellum. High-powered field evaluation (*n* = 5). Data are shown as means ± SD, *p < 0.05, one-way ANOVA with Bonferroni correction. Images shown at 10×.

Microglia are specialized macrophages that are activated by induced brain injury (9, 22). To determine the protective effect of butyrate in the neonatal brain, we first examined the population changes of microglia in different regions of the neonatal brain. Experimental NEC resulted in a greatly increased expression of the microglial marker ionized calcium-binding adaptor molecule 1 (IBA1) in the cortex, hippocampus, and cerebellum (Figure 1). Interestingly, butyrate treatment decreased NEC-induced microglia activation in several brain regions (Figure 1), especially in the cerebral cortex and hippocampus. Because activated microglia would result in a dysregulation of oligodendrocyte development (22, 23), we next examined the population of maturing oligodendrocytes. CNPase was used as the oligodendrocyte marker as it recognizes progenitors and both mature and immature oligodendrocytes (24). Consistently, NEC led to significant decreases in the oligodendrocyte population in the cerebral cortex, hippocampus, and cerebellum. Similar to microglia, butyrate treatment attenuated the decrease in the oligodendrocyte population noted with NEC in all three regions of the brain (Figure 2).

### Protective effect of butyrate on brain morphology and neural cell proliferation

**Figure 3:**
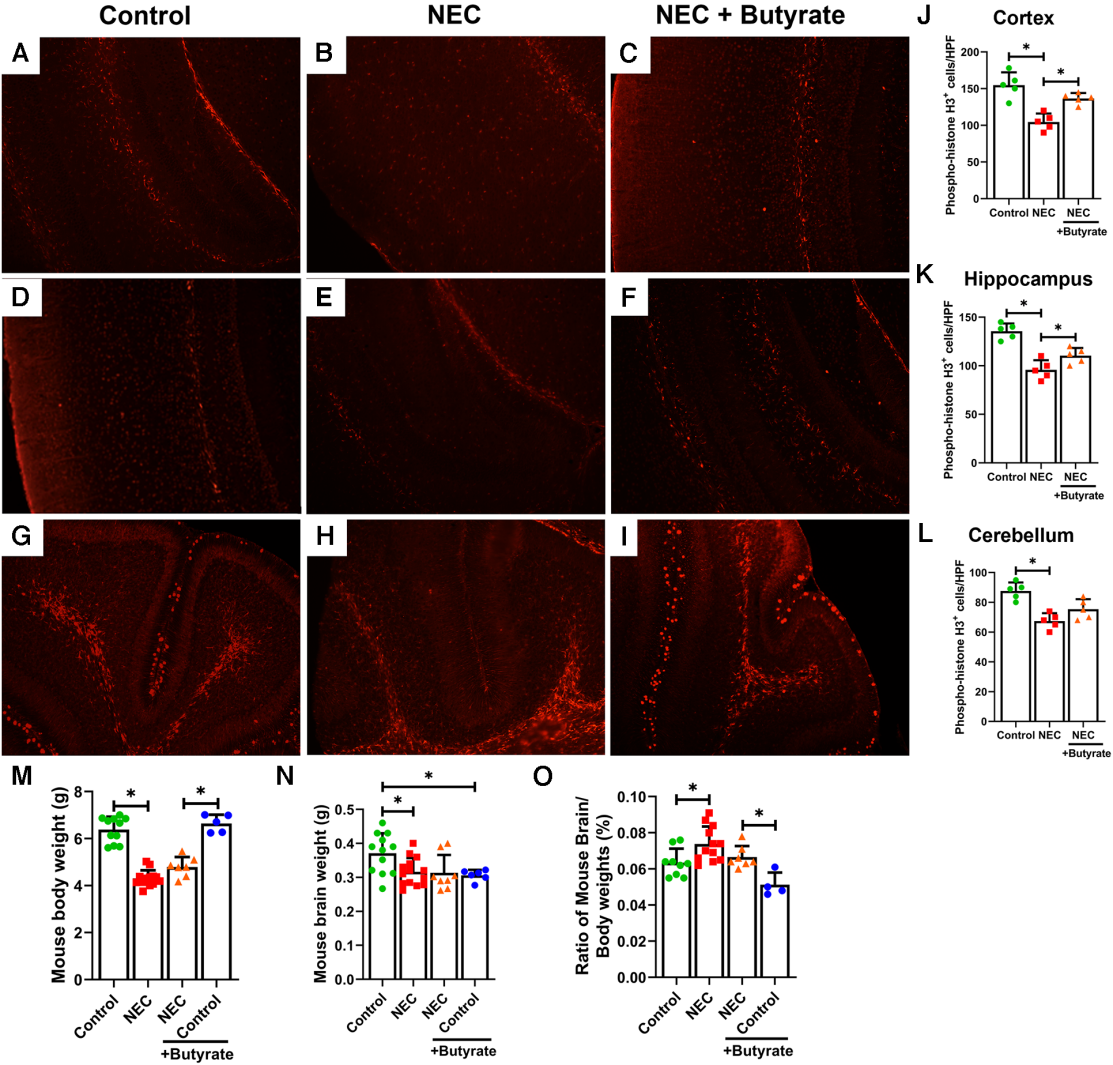
Immunofluorescence studies evaluating cell proliferation in specific regions of a developing mouse brain. Neonatal mice were treated with enteral butyrate between P5 and P7, following which experimental NEC was induced between P7 and P10. Cortex, cerebellum, and hippocampal sections were used for Phospho-Histone H3 immunofluorescence studies. Three different regions of the brain were evaluated for replicating cells in the (**A**) mouse control cortex; (**B**) mouse NEC cortex; (**C**) mouse NEC + butyrate cortex; (**D**) mouse control hippocampus; (**E**) mouse NEC hippocampus; (**F**) mouse NEC + butyrate hippocampus; (**G**) mouse control cerebellum; (**H**) mouse NEC cerebellum; (**I**) mouse NEC + butyrate cerebellum. Cell counts for the respective regions comparing the control vs. NEC vs. NEC plus butyrate group: (**J**) cortex; (**K**) hippocampus; and (**L**) cerebellum. (**M–O**) indicate brain and body weight quantifications, with (**M**) indicating total body weight, (**N**) Brain weight, and (**O**) percent brain-to-body weight. High-powered field evaluation (*n* = 5). Data shown as means ± SD, *p < 0.05, one-way ANOVA with Bonferroni correction. **p < 0.05, Kruskal–Wallis with Dunn's correction. Images shown at 10×.

The brain of neonatal mice with NEC was smaller and weighed less (305 ± 27 mg) than that of the control pups (390 ± 49 mg, *p *< 0.05) (Figure 3N). Moreover, the percent brain/body weight ratio of the NEC pups was higher (7%) than that of control pups (6%, *p *< 0.05) (Figure 3O). The loss of brain weight in the NEC group suggests that brain cell proliferation may be suppressed. Phosphorylated histone H3, a cell proliferation marker (25), was significantly decreased in the NEC group compared with the control group (except in the cerebellum). While brain weight in the NEC + butyrate group was not substantially restored when compared with the NEC group, butyrate administration helped rescue cell proliferation deficits induced by NEC (Figures 3A–L).

### Butyrate reduces NEC-induced proinflammatory signaling in the cortex and cerebellum

**Figure 4:**
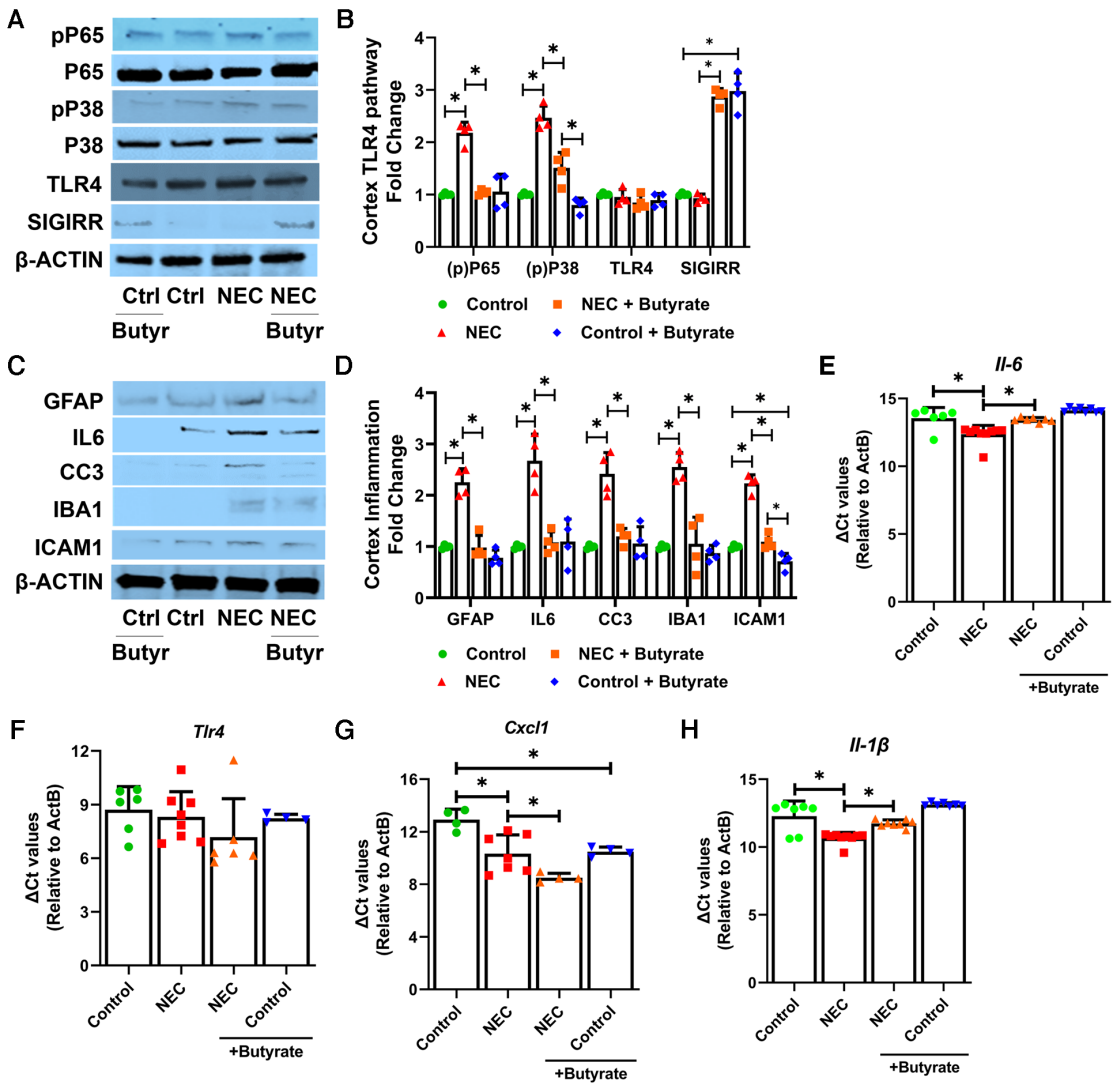
SCFAs decrease inflammatory response in the cortex region after experimental NEC. Neonatal mice were treated with enteral butyrate between P5 and P7, followed by experimental NEC induction between P7 and P10. P10 cerebral cortical samples collected on P10 were used to quantify protein and RNA expression. (**A–D**) Western blot analysis of cortex tissue lysates using indicated antibodies, with densitometry shown. *n* = 4. (**E–H**) Gene expression was quantified by real-time PCR in cortex tissue lysates (*n* ≥ 4). Data shown as means ± SD, *p < 0.05, one-way ANOVA with Bonferroni correction.

TLR4 signaling is considered a key signaling pathway in NEC pathogenesis (26). Therefore, we examined elements of TLR signaling in the cerebral cortex. The markers of canonical TLR signaling, such as phosphorylated P65 (RELA, NFκB subunit) and phosphorylated P38 MAPK, were both elevated in the NEC group but repressed with butyrate pretreatment, although the expression of TLR4 was not altered (Figures 4A,B). Previously, we have reported that SCFAs induce the intestinal expression of SIGIRR (11), a major inhibitor of TLR signaling (11). Butyrate promoted SIGIRR expression in the cortex (Figures 4A,B). Butyrate treatment attenuated NEC-induced TLR-mediated inflammation, which was evident by a decreased expression of proinflammatory cytokine interleukin 6 (IL-6), cytokines C-X-C motif chemokine ligand 1 (*Cxcl1*), and proinflammatory cellular adhesion molecule ICAM1 either at the protein or at the mRNA level (Figure 4). Cleaved caspase 3 (CC3), a marker of apoptosis, was elevated in NEC but suppressed with butyrate treatment, indicating a partial rescue of cell death (Figures 4C,D). Induction of IBA1 and GFAP expression in the cerebral cortex seen with NEC was repressed with butyrate pretreatment (Figures 4C,D).

Because the cerebellum also showed morphological changes in response to NEC that were decreased by butyrate treatment, we examined TLR4 signaling and related proinflammatory gene expression in the cerebellum. Experimental NEC modestly induced the marker of activated TLR signaling phospho-P65 (Supplementary Material 2A,B). NEC-induced proinflammatory markers ICAM1, GFAP, and IL-6 were reduced by butyrate treatment at the protein level in the cerebellum (Supplementary Material 2C,D). The MRNA levels of *Il-6*, *Tlr4*, *Cxcl1*, and *1l-1b* were not altered in the cerebellum with NEC (Supplementary Material 2E,H). Butyrate treatment effectively prevented NEC-induced cell apoptosis in the cerebellum, which was evident from decreased CC3 expression. Interestingly, NEC-induced changes in the cerebellum were much more modest than the changes observed in the cerebral cortex. We also noted that SIGIRR was not as strongly induced in the cerebellum as in the cerebral cortex.

## Discussion

In preterm neonates who develop surgical NEC, over half develop cerebral palsy and significant developmental delays (3–5). Investigations into the mechanisms by which intestinal injury in neonates causes brain injury and its lasting effects on neurosensory development are being increasingly carried out with respect to NEC, as they are incompletely understood (5, 8, 11, 27, 28). In addition, whether SCFAs impact NEC-induced brain injury remains to be defined and was the focus of our study. In this study, we show that experimental NEC induced brain injury in several areas of the brain, with the cerebral cortex being more sensitive to injury. We noted NEC-induced apoptosis, decreased proliferation, activated microglia, reduction in the number of mature oligodendrocytes, and activated proinflammatory TLR signaling in several areas of the developing brain. Enteral butyrate supplementation attenuated inflammation and cellular changes resulting from experimental NEC in association with increased cerebral cortical SIGIRR expression and decreased TLR signaling. This is one of the first studies to show that enteral butyrate treatment reduces the severity of experimental NEC-induced brain injury.

Our results show that NEC triggers pathological changes in the developing cerebral cortex, hippocampus, and cerebellum with an elevated level of microglial activation. Microglia play a pivotal role in regulating inflammation, preserving tissue equilibrium, coordinating repair processes, and influencing brain development (29, 30). The myelination of neurons, involving the sheathing of nerve fibers with a fatty coating, significantly boosts the velocity and effectiveness of neural signal transmission, making it a pivotal element in the central nervous system (CNS) development of neonates (31). Oligodendrocytes, as the primary producers of this myelin, construct a comprehensive membrane that tightly enwraps axons. This energy-intensive process, marked by a high metabolic turnover, renders oligodendrocytes susceptible to cytotoxic and excitotoxic factors (24). In the initial year of life, the white matter volume of the human brain expands by 6%–16%. Central to this growth is the process of myelination. Swift myelin development in the neocortical regions may establish the biological foundation for enhanced functionality but also render it more susceptible to pathogenic stimuli (32, 33). Microglia were greatly increased in the cerebral cortex, hippocampus, and cerebellum, indicating that brain injury induced by NEC stimulated reparative responses. NEC also decreased the oligodendrocyte population, which generates myelin that is required for normal neural signaling (23, 24). Our results are consistent with previous findings showing a decrease in the number of oligodendrocytes and replicating cells with experimental NEC (8–10). We then focused on the inflammatory response as NEC induces intestinal inflammation and, in severe cases, systemic inflammation. We found that NEC induced the expression of inflammatory cytokines IL6, CXCL1, and IL1β in the cerebral cortex, correlating with an increased activation of canonical TLR signaling pathways such as phospho-P65 and phospho-P38 MAPK. The activation of TLR signaling in the brain could have resulted from an impaired blood–brain barrier and bacterial entry or from a systemic activation of damage-associated molecular patterns that can signal through TLRs (9, 34, 35). Interestingly, we noticed that the cerebellum showed less prominent TLR signaling activation and inflammation compared with the cerebral cortex. This might represent differences in the vulnerability of different regions of the brain to NEC-induced brain injury. Consistent with our studies showing a more prominent injury in the cortex, studies in preterm infants also show defects in myelination, cerebral cortical injury, and periventricular leukomalacia prominently in the cortex as against the cerebellum, contributing to cerebral palsy (3, 6, 36, 37). As we studied inflammation several days after NEC induction, it is possible that early cytokine induction seen with NEC was not captured by our data.

Short-chain fatty acids, especially butyrate, have been noted to play an important role in intestinal inflammation and maintaining homeostasis (38–40). Previous studies have shown that premature infants have decreased intestinal concentrations of SCFAs when compared with term infants (13). The decreased concentrations of SCFAs could serve as a potential risk factor for the development of NEC and NEC-induced brain injury. We have reported that enteral butyrate represses proinflammatory TLR signaling and intestinal injury after experimental NEC in mice in parallel with the induction of SIGIRR and A20, major inhibitors of TLR4 signaling (11). We found that enteral butyrate treatment attenuated NEC-induced brain injury in neonatal mice. More importantly, the activation of TLR signaling, as evident from the expression of phospho-P65, phospho-P38 MAPK, and the cytokines *Il6* and *Icam1*, decreased with butyrate treatment prominently in the cerebral cortex. The decreased number of activated microglia with butyrate treatment is consistent with decreased inflammation. Decreased CC3 levels and a smaller decrease in the oligodendrocyte population and cell proliferation with butyrate treatment indicated that NEC-induced cellular toxicity was decreased by enteral butyrate. Interestingly, the expression of SIGIRR, a strong inhibitor of TLR signaling, was induced with butyrate treatment in the cerebral cortex, similar to what we reported in the newborn intestine (11). A strong induction of SIGIRR in the brain, in association with decreased TLR signaling, suggests that butyrate directly or indirectly activates the anti-inflammatory responses in the brain. Previous work has shown that butyrate treatment reduced systemic LPS-induced microglia activation and depression-like behaviors in adult mice, although SIGIRR was not directly examined (41). Our data revealing improvements in cell proliferation with butyrate are consistent with those in previous work showing that SCFAs increase the proliferative potential of human neural progenitor cells *in vitro* (42).

Butyrate potentially exerts its effects on suppressing NEC-induced brain inflammation and injury through multiple pathways. Initially, it directly hinders the activity of histone deacetylases, which play a crucial role in neuroinflammation (43). Recent findings also suggest that butyrate directly stimulates the p300 histone acetyltransferase (44). Both activities lead to increased histone H4 acetylation at the promoters of inflammatory cytokines and NFκB P65, resulting in a diminished expression of inflammatory genes. In addition, butyrate may operate through the production of ketone bodies, particularly R-β-hydroxybutyrate, to safeguard against NEC-induced brain injury. This may involve butyrate's ability to enhance the generation of ketone bodies (45), which are known to be involved in both neuroinflammation and neuroprotection (46). Butyrate-mediated intestinal barrier enhancement may also contribute to the suppression of systemic inflammation (40, 47).

This study has specific limitations. Initially, there is a possibility that butyrate was absorbed and directly influenced the CNS; however, we did not measure changes in butyrate concentration in the blood and terminal ileum after oral administration. While we observed the induction of SIGIRR, inhibitors of TLR signaling, in the brain, we did not show whether this could be mediated via butyrate absorbed in the intestine. Alternatively, butyrate may induce a metabolite in the gut that can exert its protective effects in the brain by inducing SIGIRR. Demonstrating an increase in the systemic or brain levels of butyrate and assessing its impact on systemic cytokines would provide clarity on this matter. Second, a recent study by Zhou et al. revealed that CD4+ T cells derived from the gut can travel to the brain and cause brain injury (9). Butyrate's potential effect on T cell–mediated cytotoxicity in NEC–related brain injury was not examined in this study (9). Third, microglial cell polarization plays a direct role in neuroinflammation (48). In a study examining alcohol-induced chronic brain injury, Wei et al. demonstrated that intervention with butyrate effectively restored the disrupted balance of microglial M1/M2 polarization (49). Further exploration of the microglial M1/M2 phenotype in the context of NEC and NEC with butyrate treatment in the brain could yield valuable insights and expand our understanding of the potential effects of butyrate.

Our study reveals the protective role of butyrate, a short-chain fatty acid, in a mouse model of NEC-induced brain injury. While we noted that the cerebral cortex was particularly vulnerable to experimental NEC injury, butyrate administration led to a broad reduction in inflammatory cytokine expression along with a partial rescue of apoptosis and cell proliferation in several cell subtypes in the developing brain. The protective effects of butyrate shown in our study are consistent with those in emerging literature showing the effect of gut microbiota and microbiota-derived metabolites on brain development, and how disruption of the gut–brain axis has long-term consequences for brain development (27, 28, 50). Future studies will focus on the impact of butyrate on rescuing long-term neurodevelopmental behavior impairments associated with NEC in mice and dissecting direct vs. indirect effects of butyrate on the gut–brain axis and on neonatal brain injury. Our results highlight the therapeutic potential of butyrate in NEC-induced brain injury and suggest that enteral supplementation with short-chain fatty acids or probiotics that can increase gut luminal concentrations of SCFAs might afford protection against NEC-induced brain injury.

## Data Availability

The original contributions presented in the study are included in the article/**Supplementary Material**, further inquiries can be directed to the corresponding author.
