# Supplementary material for: Butyrate suppresses experimental necrotizing enterocolitis–induced brain injury in mice
Source: Front Pediatr. 2023 Dec 7;11:1284085. doi: 10.3389/fped.2023.1284085 (PMC10733464; doi:10.3389/fped.2023.1284085)
Supplement: Supplementary file 1 [file Datasheet1.pdf]

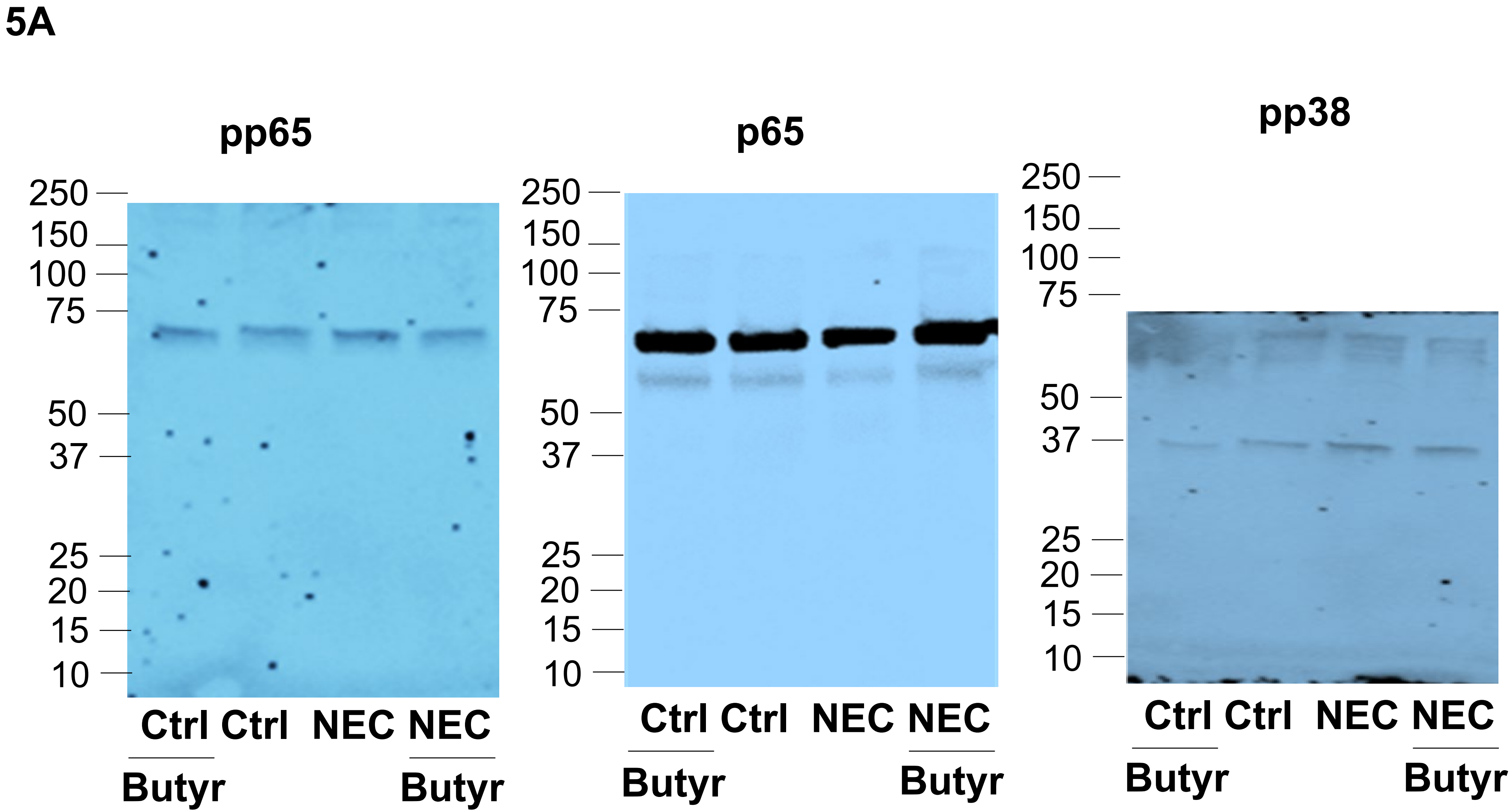
5A
pp65
250
150
100
75
50
37
25
20
15
10
Ctrl Ctrl NEC NEC
Butyr
Butyr
p65
250
150
100
75
50
37
25
20
15
10
Ctrl Ctrl NEC NEC
Butyr
Butyr
pp38
250
150
100
75
50
37
25
20
15
10
Ctrl Ctrl NEC NEC
Butyr
Butyr

5A

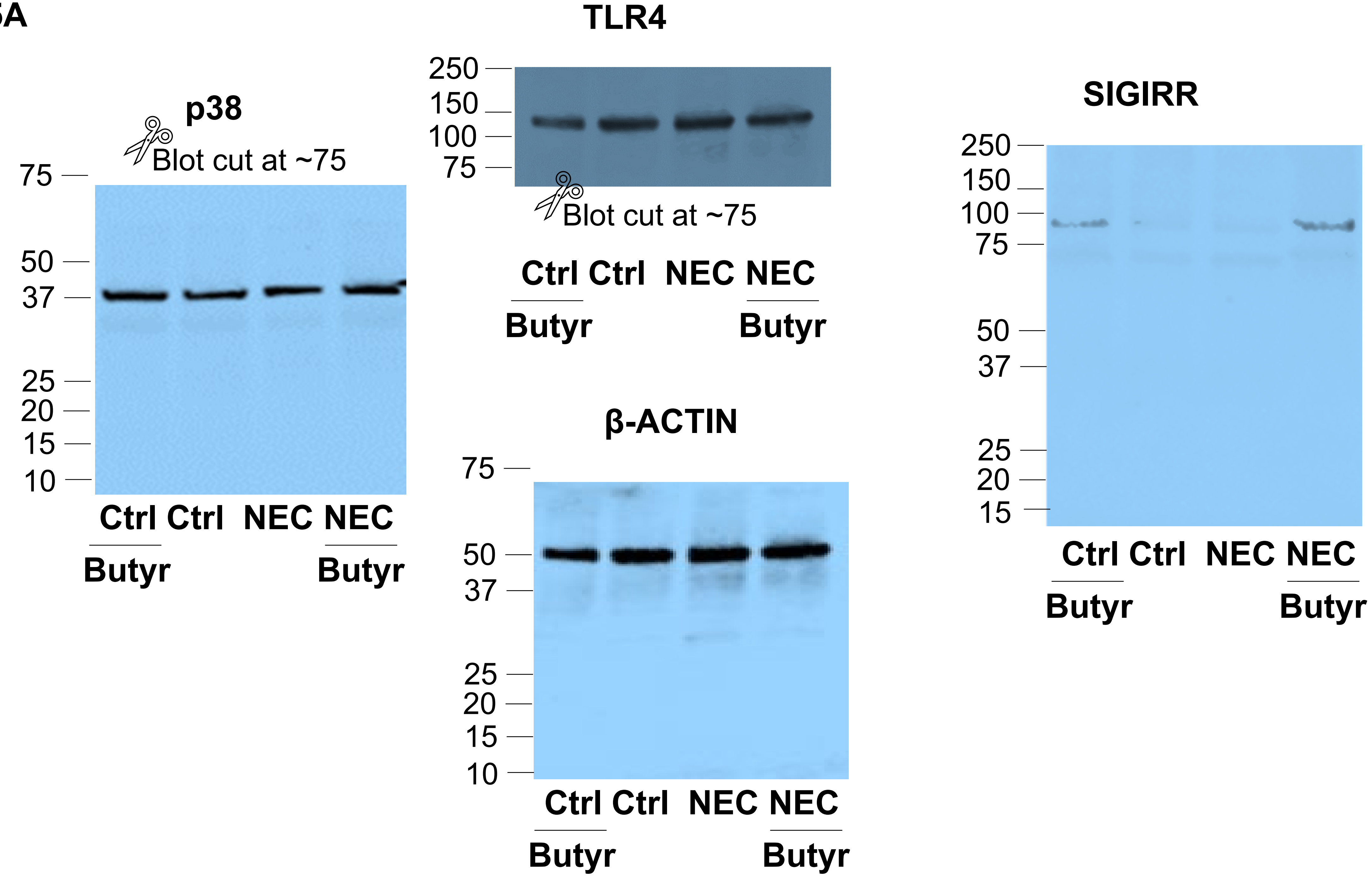

p38
Blot cut at ~75
75
50
37
25
20
15
10
Ctrl Ctrl NEC NEC
Butyr
Butyr
TLR4
250
150
100
75
Blot cut at ~75
Ctrl Ctrl NEC NEC
Butyr
Butyr
β-ACTIN
75
50
37
25
20
15
10
Ctrl Ctrl NEC NEC
Butyr
Butyr
SIGIRR
250
150
100
75
50
37
25
20
15
Ctrl Ctrl NEC NEC
Butyr
Butyr

5C

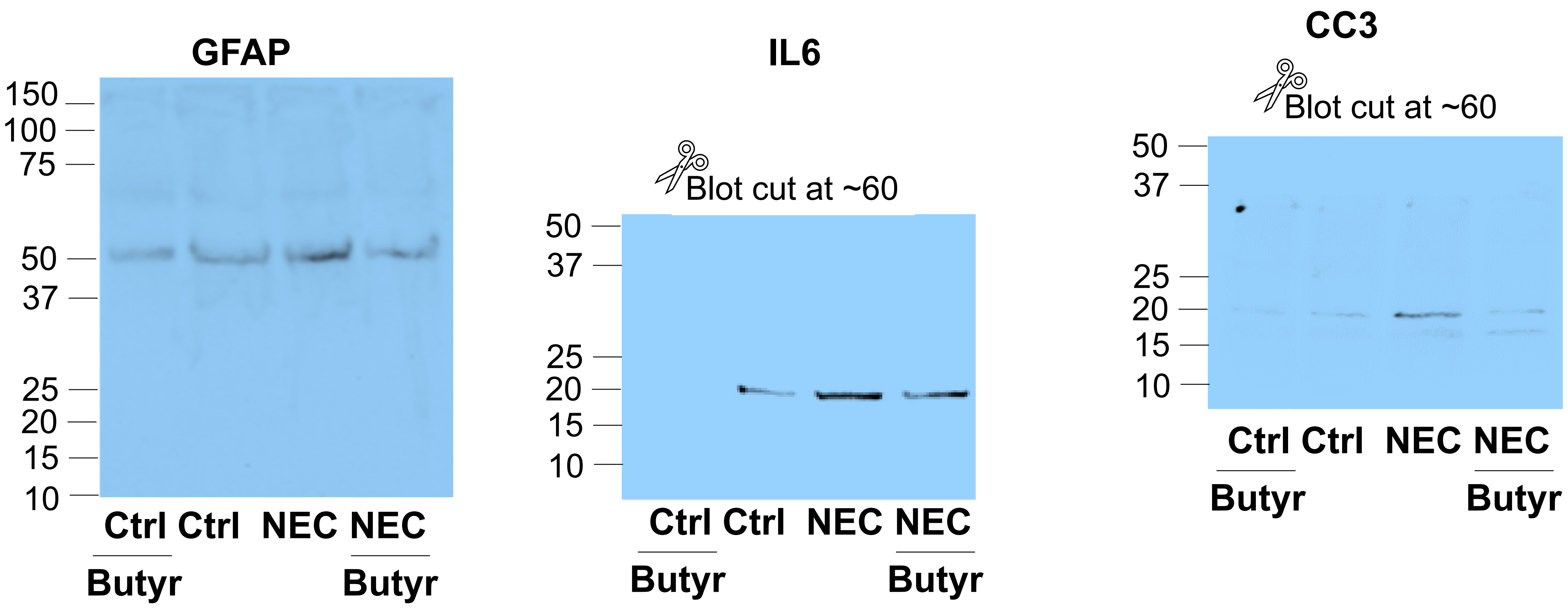

GFAP
150
100
75
50
37
25
20
15
10
Ctrl Ctrl NEC NEC
Butyr
Butyr
IL6
Blot cut at ~60
50
37
25
20
15
10
Ctrl Ctrl NEC NEC
Butyr
Butyr
CC3
Blot cut at ~60
50
37
25
20
15
10
Ctrl Ctrl NEC NEC
Butyr
Butyr

5C

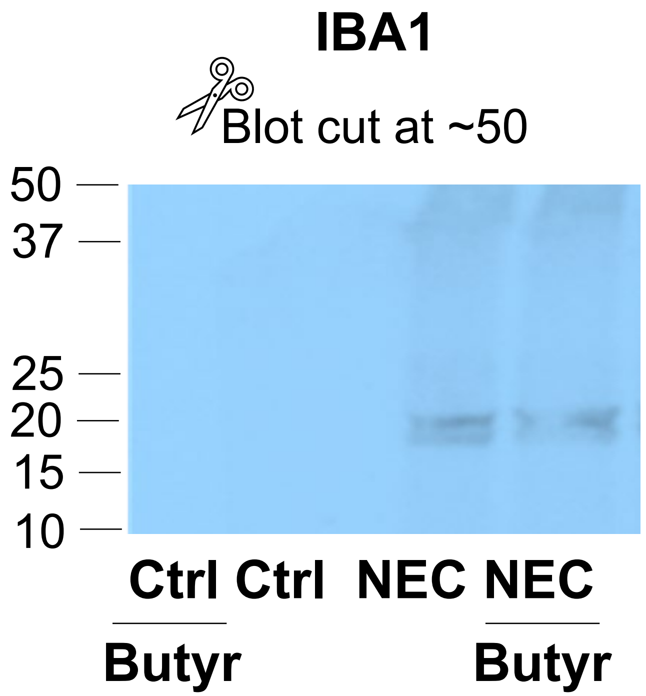

IBA1
Blot cut at ~50
50
37
25
20
15
10
Ctrl Ctrl NEC NEC
Butyr
Butyr

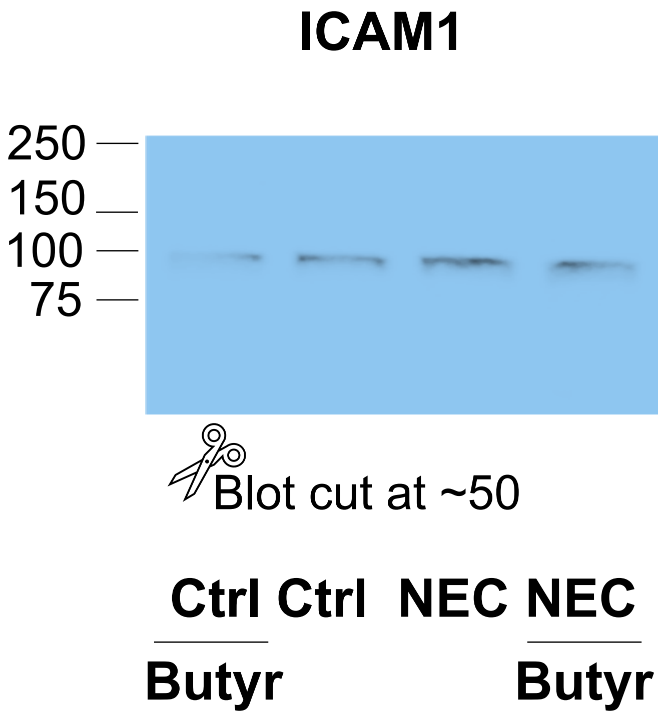

ICAM1
250
150
100
75
Blot cut at ~50
Ctrl Ctrl NEC NEC
Butyr
Butyr

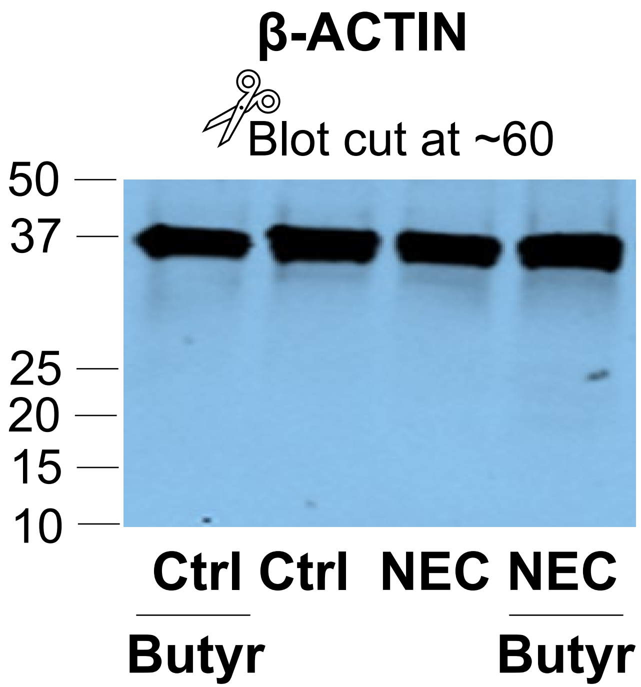

β-ACTIN
Blot cut at ~60
50
37
25
20
15
10
Ctrl Ctrl NEC NEC
Butyr
Butyr

6A

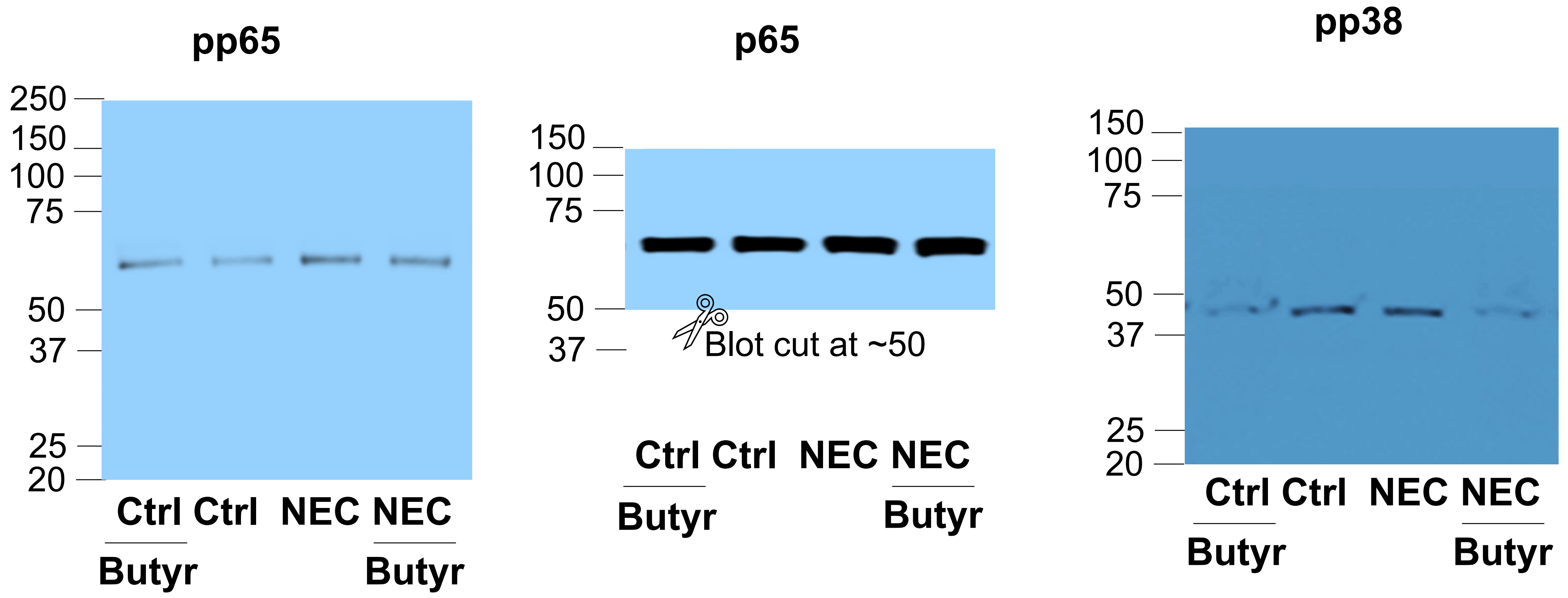
pp65
250
150
100
75
50
37
25
20
Ctrl Ctrl NEC NEC
Butyr
Butyr
p65
150
100
75
50
37
Blot cut at ~50
Ctrl Ctrl NEC NEC
Butyr
Butyr
pp38
150
100
75
50
37
25
20
Ctrl Ctrl NEC NEC
Butyr
Butyr

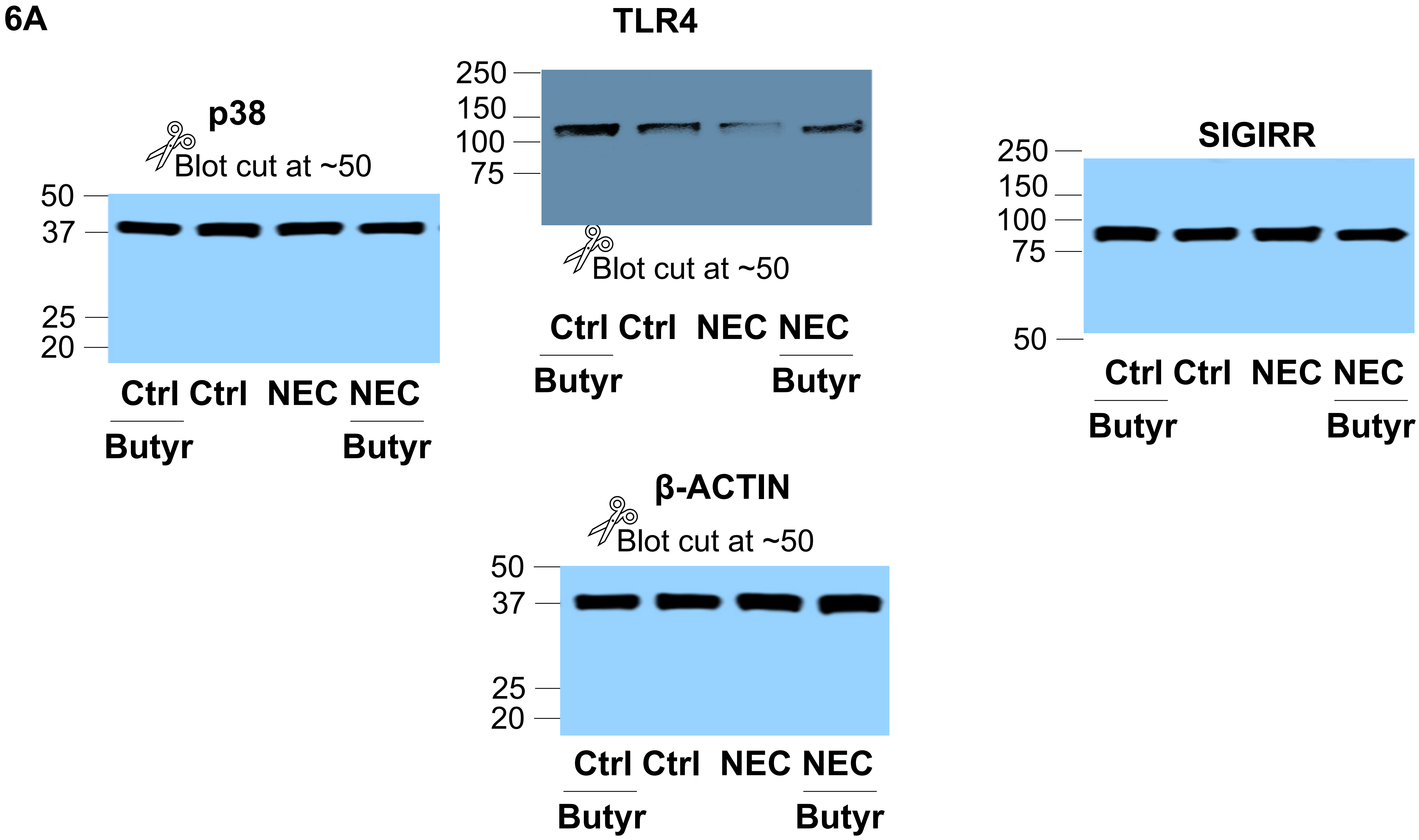
6A
TLR4
250
150
100
75
Blot cut at ~50
Ctrl Ctrl NEC NEC
Butyr
Butyr
p38
Blot cut at ~50
50
37
25
20
Ctrl Ctrl NEC NEC
Butyr
Butyr
SIGIRR
250
150
100
75
50
Ctrl Ctrl NEC NEC
Butyr
Butyr
β-ACTIN
Blot cut at ~50
50
37
25
20
Ctrl Ctrl NEC NEC
Butyr
Butyr

6C

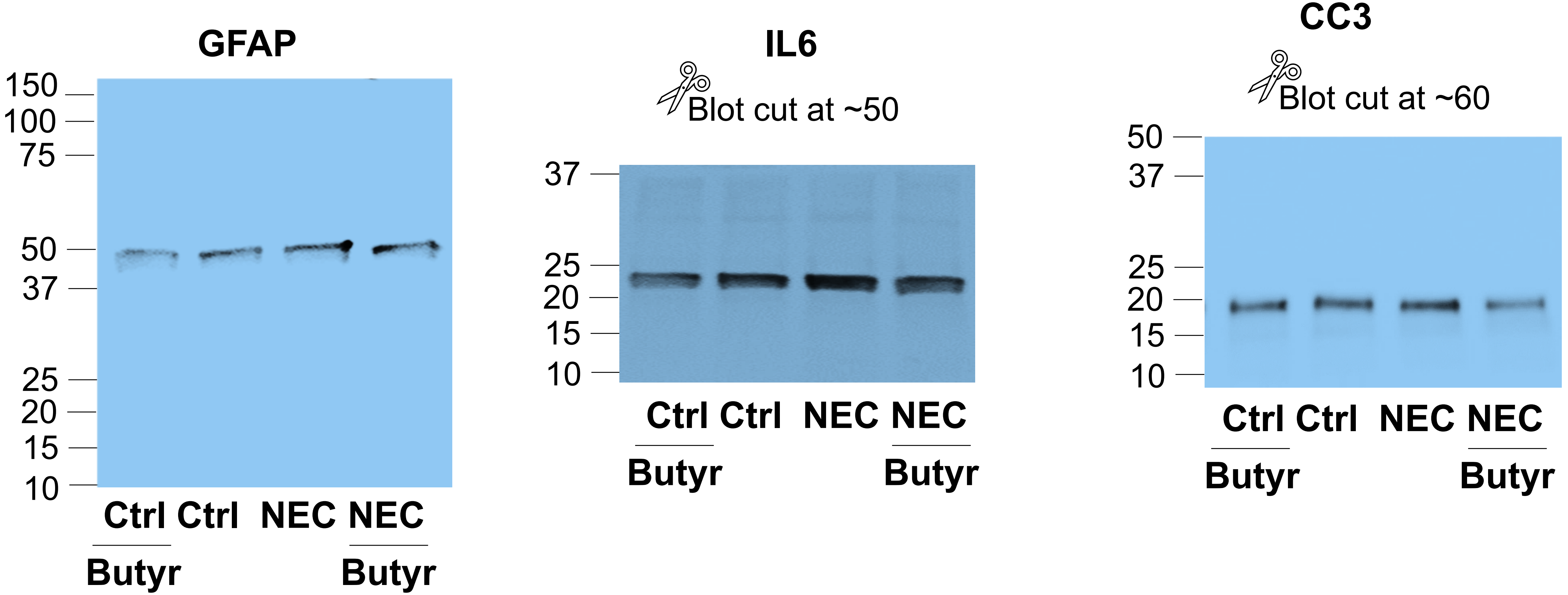
GFAP
150
100
75
50
37
25
20
15
10
Ctrl Ctrl NEC NEC
Butyr Butyr
IL6
Blot cut at ~50
37
25
20
15
10
Ctrl Ctrl NEC NEC
Butyr Butyr
CC3
Blot cut at ~60
50
37
25
20
15
10
Ctrl Ctrl NEC NEC
Butyr Butyr

6C

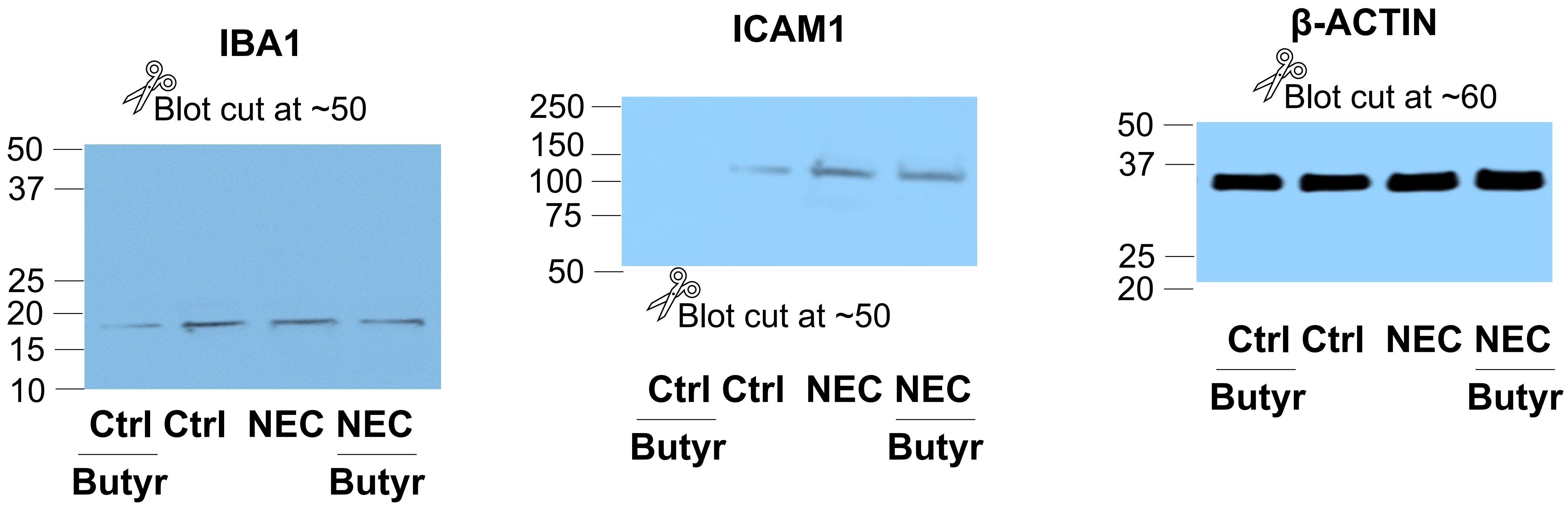
IBA1
Blot cut at ~50
50
37
25
20
15
10
Ctrl Ctrl NEC NEC
Butyr
Butyr
ICAM1
250
150
100
75
50
Blot cut at ~50
Ctrl Ctrl NEC NEC
Butyr
Butyr
β-ACTIN
Blot cut at ~60
50
37
25
20
Ctrl Ctrl NEC NEC
Butyr
Butyr
