# Supplementary figures and images for: Butyrate suppresses experimental necrotizing enterocolitis–induced brain injury in mice
Source: Front Pediatr. 2023 Dec 7;11:1284085. doi: 10.3389/fped.2023.1284085 (PMC10733464; doi:10.3389/fped.2023.1284085)

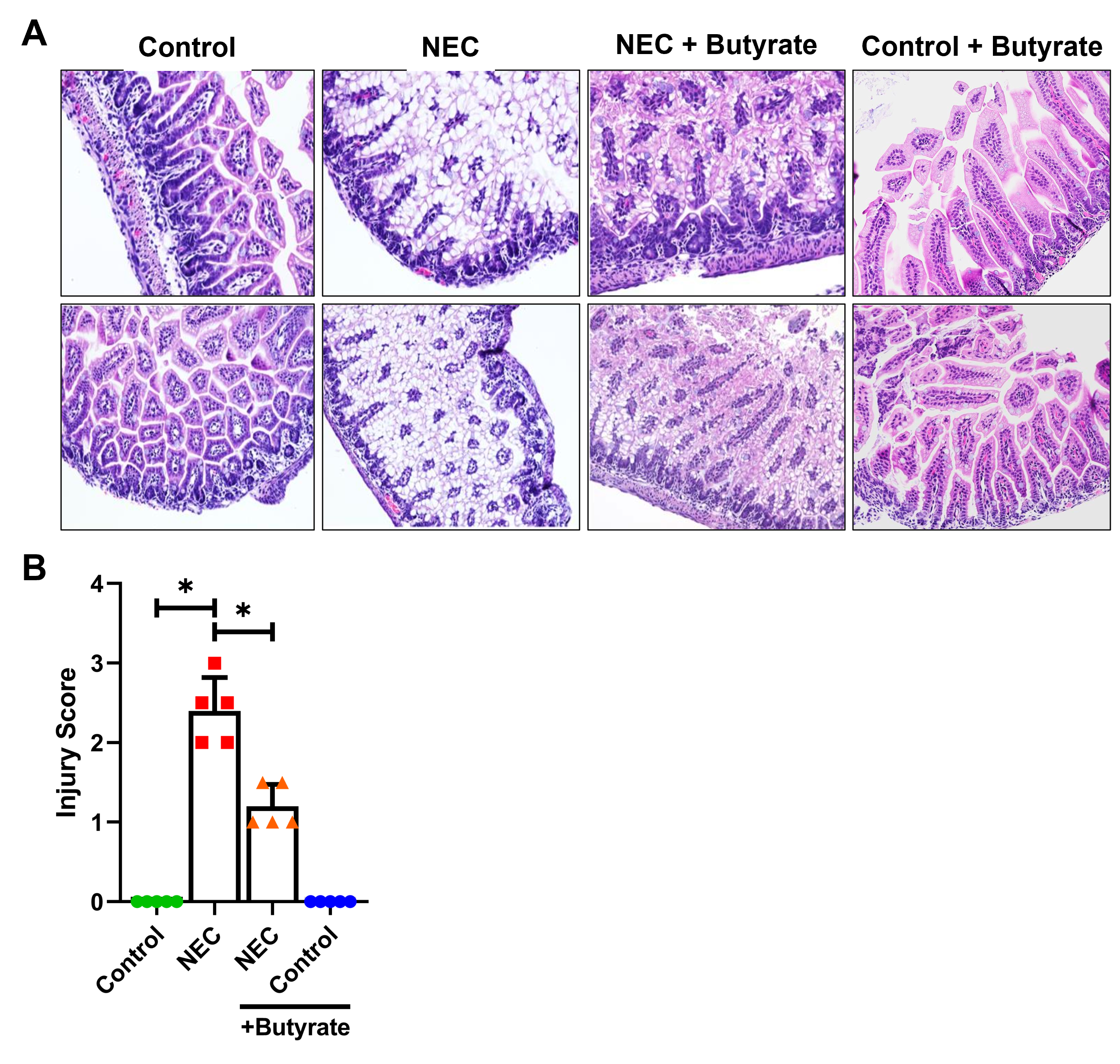

Supplement: Supplementary file 2 [file Image1.tif]

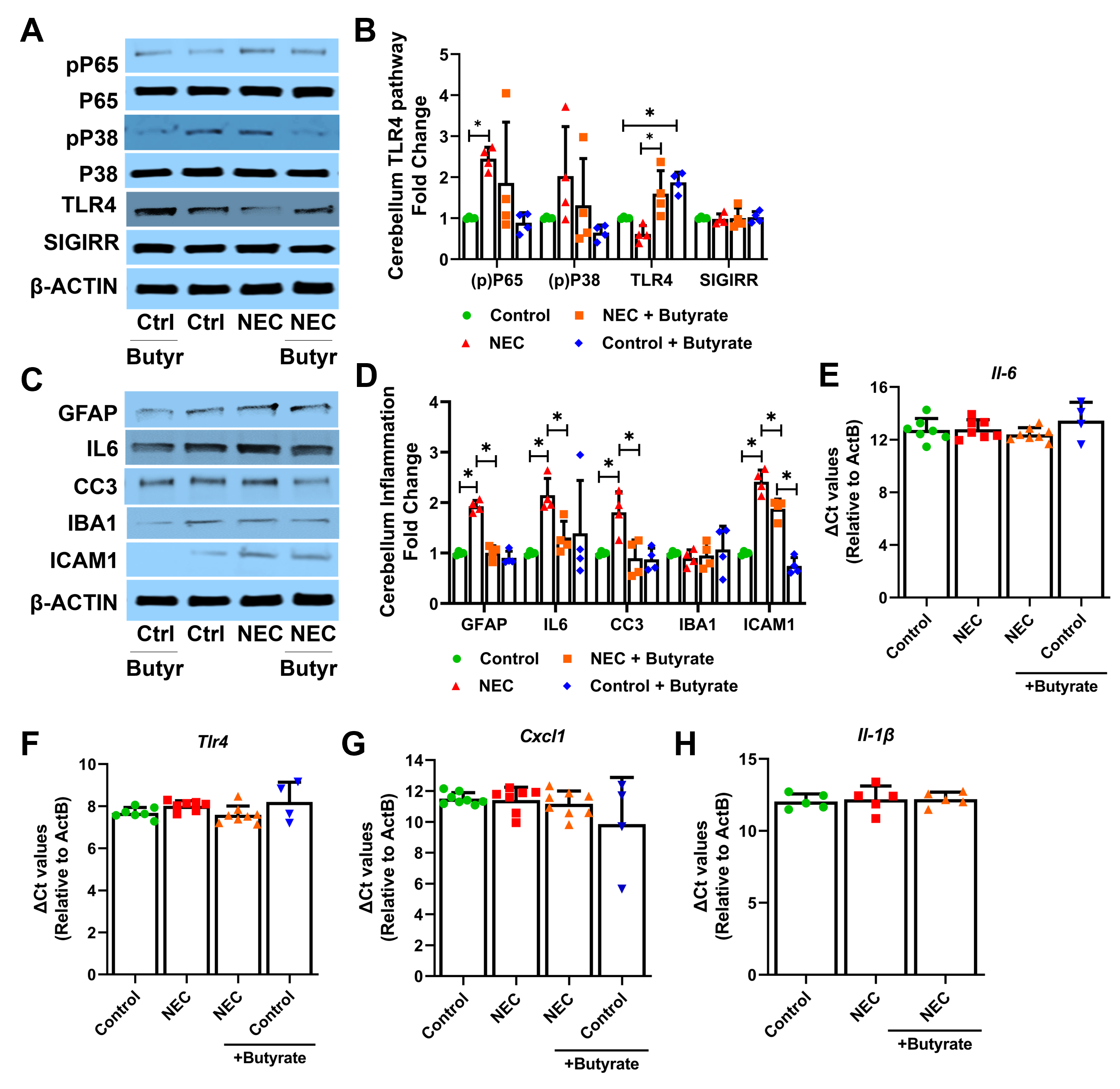

Supplement: Supplementary file 3 [file Image2.tif]

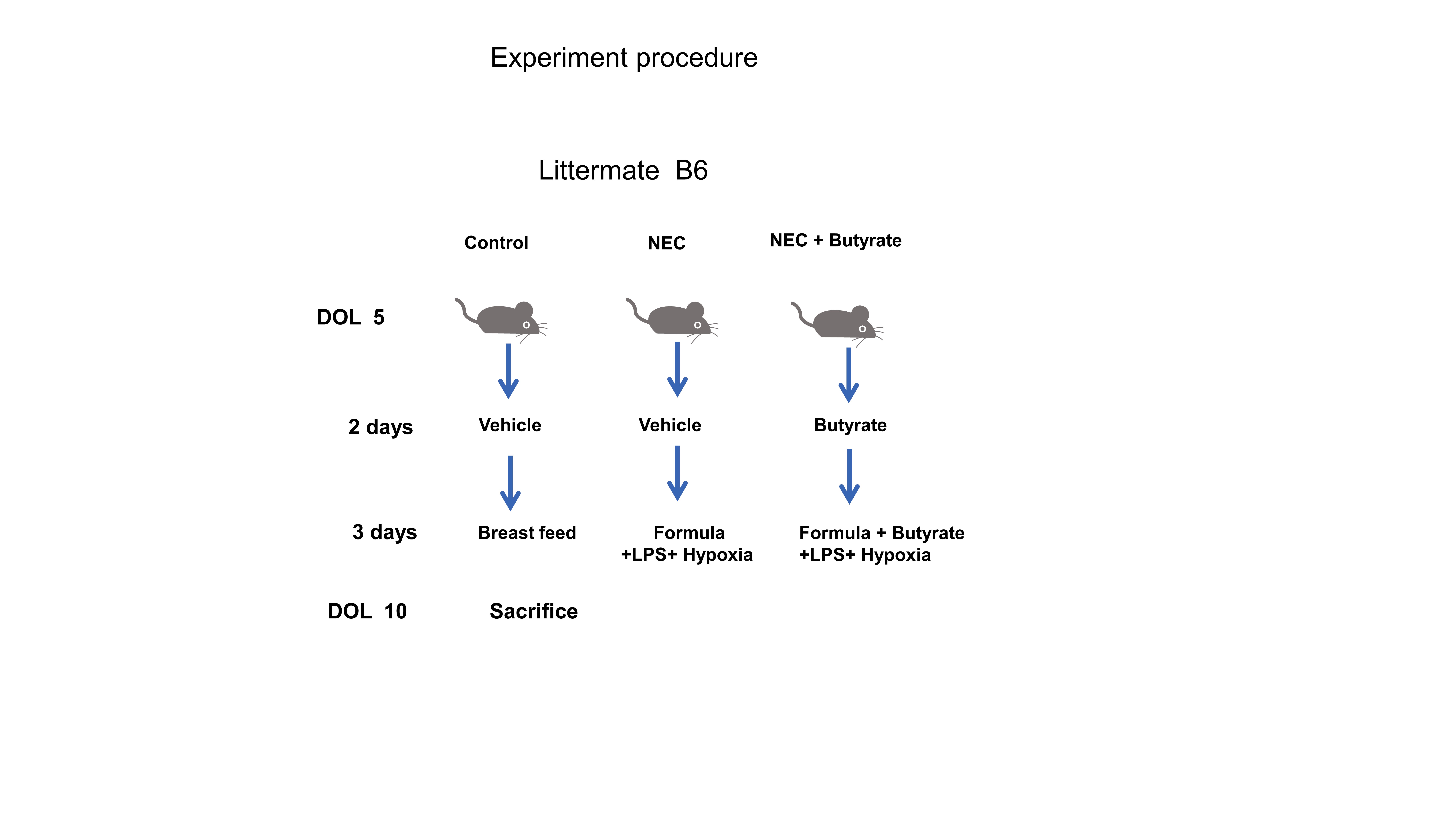

Supplement: Supplementary file 4 [file Image3.jpeg]
